# Prevalence of obesity and overweight in adults and children in Iran; a systematic review

**DOI:** 10.1186/s40200-014-0121-2

**Published:** 2014-12-23

**Authors:** Shahrzad Jafari-Adli, Zahra Jouyandeh, Mostafa Qorbani, Ahmadreza Soroush, Bagher Larijani, Shirin Hasani-Ranjbar

**Affiliations:** Obesity and Eating Habits Research Center, Endocrinology and Metabolism Molecular-Cellular Sciences Institute, Tehran University of Medical Sciences, Tehran, Iran; Department of Community Medicine, Alborz University of Medical Sciences, Karaj, Iran; Non communicable Diseases Research Center, Endocrinology and Metabolism Population Sciences Institute, Tehran University of Medical Sciences, Tehran, Iran; Endocrinology and Metabolism Research Institute, Tehran University of Medical Sciences, 5th floor of Shariati Hospital, North Karegar St., P.O Box 1411413137, Tehran, Iran

**Keywords:** Obesity, Overweight, Prevalence, Iran, Metabolic syndrome

## Abstract

**Background:**

Obesity is one of the most important underlying risk factors for chronic disease. Dramatically increasing and following complication of obesity should be alerted to health politicians and practitioners to prevent associated health risks. This review aimed to give a better insight into the prevalence of obesity and overweight in different areas of Iran.

**Method:**

All published internal (SID, Irandoc, Iranmedex), and international (Web of Knowledge, Pubmed, Scopus) source studies, reported the prevalence of overweight/obesity among normal population samples, during Jan 2005 through Jan 2014, were assessed in this review. Paper selection processes were done by two researchers separately. Studies which met the eligible criteria were included in this review.

**Result:**

One hundred ninety three eligible studies enter into our review. Of 193 final selected studies, 86 (15 national, 71 sub national) of them were reported the prevalence of obesity/overweight in adult, and 107 studies (11 national, 96 sub national) in under-18 by individual. The range of overweight and obesity prevalence in national studies in adult, was 27.0-38.5 (95% CI: 26.8-27.1, 37.2-39.8), and 12.6-25.9 (95% CI: 12.2-13.0, 24.9-26.8), separately. In under-18 the range of overweight and obesity prevalence in national studies were 5.0-13.5 (95% CI: 4.5-5.5, 13.4-13.6), and 3.2-11.9 (95% CI: 3.0-3.4, 11.3-12.4).

**Conclusion:**

Obesity as an important public health problem has been discussed in recent few decades worldwide. Although the national reported prevalence of obesity in Iran was not considerably diverse, but remarkable differences were seen in the sub national prevalence which must be noticed more in political health programs especially among women and children.

**Electronic supplementary material:**

The online version of this article (doi:10.1186/s40200-014-0121-2) contains supplementary material, which is available to authorized users.

## Introduction

Obesity as an epidemic of 21st century is a major public health problem worldwide which its prevalence is dramatically increasing in both developed and developing countries [1,2]. Obesity or overweight are physiologically defined as fat accumulation in an abnormal or excessive pattern in adipose tissue may cause some serious health concerns [3].

The Body Mass Index (BMI) is the most prevalent and practical indicator for evaluation of overweight and obesity in adults around the world [4], based on it, individuals with BMI 25–30 kg/m^2^ and >30 are defined as overweight and obese, respectively [5]. Although there is no general agreement on a cutoff point of overweight or obesity in children and adolescents, International Obesity Task Force (IOTF) and The US Centers for Disease Control and Prevention (CDC), are more acceptable [6].

Obesity is a serious health problem with side effects can vary from a complaint of disability to premature death. So decreasing in quality of life (QOL) is unavoidable [7,8]. Some studies have proclaimed that maybe increasing rate of obesity prevalence is associated with the notable changes in the life style especially dietary habits (consumption of unhealthy outdoor foods) and inadequate daily activity in both rural and urban regions [3,9]. Genetic factors; different pattern of eating; and low socioeconomic status are some of the ethnic dimensions that can be underlying basis for obesity as well [10-13].

Overweight is the sixth most important risk factor causative to the total worldwide disease [14]. Some studies found that obesity increases the risk of some chronic and life threatening disorders such as type 2 diabetes, Cardiovascular disease, hypertension, hyperlipidemia, sleep apnea, and followed by, it has been estimated to reduce life expectancy around 7 years [15-17]. So researchers are always looking for appropriate treatment methods for obesity [17,18].

Along with, we will be faced a big health problem by growing demands in based on obesity co-morbidity disorders that definitely required to spend huge funds and limitation resources in the near future [10,19].

Based on global evaluations by WHO in 2005, the number of overweight and obese individuals throughout the world reach to 1.6 and 400 million person respectively [5,20], and expected to get to 2-3 billion overweight and more than 700 million obese in 2015. Studies revealed that the prevalence of overweight and obesity in most Asian countries as well [4,10].

We conducted this literature review to evaluate the prevalence of obesity and overweight in Iran with particular attention to differences living classes (urban, rural), type of study (national, sub-national), gender (male, female) in two age categories (adult, under-18-years-old).

## Methods

In this systematic review we endeavored to assess all the related studies that report prevalence of obesity and overweight in all regions of Iran. The detail of this study strategy will be mentioned in following.

### Search strategy

Electronic search strategies were designed by an experienced medical information specialist in consultation with the review team. Search strategy was assessed and accepted in a peer review board of Endocrinology and Metabolism Research Institute of Tehran University of Medical Sciences. We searched in English databases; Scopus, ISI web of Sciences and PubMed and also in Persian databases; IranMedex, Scientific Information System (SID), and Irandoc to obtain all related studies, during time ranged Jan 2005 up to Jan 2014. All databases were assessed in title, keyword, and abstract.

The medical subject headings (MESH) were; “obesity”, “overweight”, “Iran”, and “prevalence” for searching in English databases and also the equivalent Persian- language of these terms were used for searching in Persian databases. Besides, hand-searching was conducted to find articles which not found in electronic search.

Forasmuch as the differences in cut of points between adult and children/adolescents, the standard cutoff points in each study were recorded. The reports for children/adolescents prevalence were based on standards CDC (The Centers for Disease Control and Prevention), WHO (World Health Organization), and IOTF (The International Obesity Task Force). CDC is based on growth curves that released new international growth standards for children aged 5 years or younger that include BMI-for-age growth charts defines obesity and overweight as higher than the 95th, and 85-95th percentile of body mass index, respectively. The National Center for Health Statistics/World Health Organization NCHS/WHO cut-Off percentile classification for age including: underweight < 5th BMI-for-age; normal weight 5th < BMI-for-age < 85th; overweight 85th BMI-for-age < 95th; and obese BMI-for-age > 95th. The same percentiles cut-offs were considered for the CDC as well. IOTF used data from six national studies carried out in different countries to provided percentile curves that passed through the widely used cut-off points of 25 kg/m^2^ and 30 kg/m^2^ for adult overweight and obesity [21].

### Study selection

We included all related population-based studies including national, province, and local surveys which were carried out on individuals with no restraint in age or gender. Extracted information of children and adults was recorded in two separate sheets of Excel software (Microsoft office package 2010) including; name of the first author, year of publication, study region, level of study (national, province, local), number of sample size (total, and sex or regional subgroups number), standard cut-off point for obesity and overweight, reported prevalence and its 95% confidence interval.

### Exclusion criteria

We excluded studies with fewer than 200 individuals, non-population-based, and non-randomized. Duplicated citation and review articles were excluded from the study. English duplicated studies were emitted by Endnote software, and Persian studies by hand.

### Data extraction

Evaluation of inclusion and exclusion criteria was conducted by assessing title and abstracts of studies in the first and second steps respectively. Then full texts were reviewed in cases which were not sufficient by reading their abstracts. As regards BMI as a conventional variable was be noted in many studies, we are proposed to study full text to extract prevalence of obesity or overweight even in cases with no main aim of determination of obesity or overweight prevalence. All procedures were performed by two researchers independently. In cases there were no agreement, the debate was conducted to achieve consensus. Otherwise the third expert reviewer was asked to decide in that case. Finally information was inserted in two separate tables for adult and under-18-individuals.

## Results

**Figure 1:**
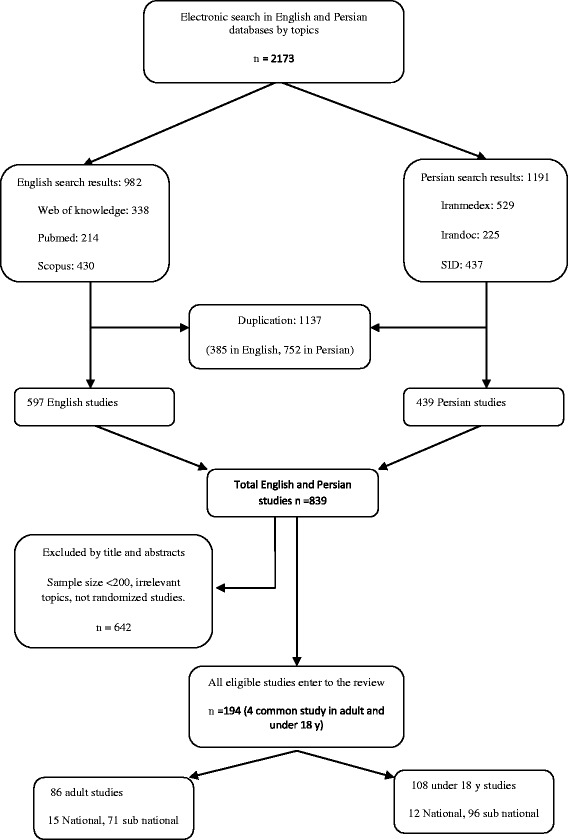
**Flow diagram of the study selection process.**

In primary search we found 2173 full text articles based on the related keywords to our topic. Of those, 982 were obtained from English data bases and the rest attained in Persian databases. After excluding overlapped studies, and considering inclusion and exclusion criteria in two separate steps (title and abstract review), finally 194 (86 for under-18 and 108 for adult) qualified studies were selected to enter to our review. The number of primary research results and the detailed process to select appropriate studies are shown in the Figure 1.

The extracted data from these studies are shown separately in 2 tables (Additional file 1: Table S1 and Additional file 2: Table S2) which include the level, location, year of publication, number of population, and prevalence of obesity and overweight in adults and under-18-years-old in Iran. Some studies reported the prevalence of obesity and overweight together, so for not losing the data, they were put on the separate columns in tables as well. In cases which were reported the prevalence in male/female or urban/Rural areas, the extracted data put in the tables separately. In view of the differences in standard diagnosis criteria for obesity and overweight in under-18-related studies, the criteria in these studies put in the table as well.

We found 15 and 12 National, and 71 and 96 sub national studies in adult and under-18 y related studies, respectively. There were found 4 common studies which carried out among adult and under-18 individuals. Considering the wide range of prevalence in different studies, to find a better overview of studies, we placed a summary of maximum and minimum reported prevalence in national and sub national studies in Additional file 3: Table S3.

The range of overweight and obesity prevalence in sub national studies in adult, were 12.8-76.4 (95% CI: (9.2-17.3, 75.1-77.6), and 2.4-35.4 (95% CI: 1.0-4.9, 31.4-39.6), respectively. The range of overweight and obesity among adult national studies were 27.0- 38.5 (95% CI: 26.8-27.1, 37.2-39.8), and 12.6-25.9 (95% CI: 12.2-13.0, 24.9-26.8). The first, second and third greatest national reported prevalence of obesity and overweight were 25.9, 25.1, and 22.3 among adult, and 11.9, 10.4, and 6.5 among under-18 population. The range of overweight and obesity in sub national studies among under-18 y were 2.4-67.1 (95% CI: 1.5-3.7, 63.3-70.8), and 0.6-27.7 (95% CI: 0.3-1.1, 25.4-30.0). The range of national reported prevalence of overweight and obesity in national studies among under-18 y were 5.0-13.5 (95% CI: 4.5-5.5, 13.4-13.6), and 3.2-11.9 (95% CI: 3.0-3.4, 11.3-12.4). The lowest and the highest prevalence of obesity in adult appertain to Shiraz and Rasht, and among under-18 y concern to Kerman and Gorgan.

## Discussion

Obesity is the most apparent demonstration of an inappropriate sedentary lifestyle and increase in high caloric food consumption [22]. In 2005, a study of global burden of obesity, reported the estimated total numbers of overweight and obesity in adults, 937 million (23.2%), and 396 million (24.0%), respectively. It were projected the number of overweight and obese people in 2030 reach to 1.35 billion and 573 million [23]. The National Health and Nutrition Examination Survey in the United States as a country with a high prevalence of obesity and overweight, in 2010, more than one third of adult were obese [24]. In another adult population study in Spain, the prevalence of obesity was 22.9% (24.4% in men and 21.4% in women) [25]. The prevalence of overweight in Turkey was 19.0% in 2007 (17.4% in men, 20.4% in women) [26]. Among Asian countries, it is reported that the prevalence of obesity was 10.3%, and the prevalence of overweight and obesity together were 25.0% in Pakistan [27]. In our review the prevalence of overweight and obesity in adult national studies were higher with range 27.0-38.5 and 12.6-25.9, respectively.

On the other hand, obesity among children and adolescents has more than doubled and quadrupled in the past 3 decades [28]. Based on NCHS data brief in 2010, almost 17% of youth were obese [24]. Obese children are more likely to suffer from obesity in adulthood. Obesity at young ages is responsible for increasing risk of obesity-related diseases in children, even some disease in the past known as adult diseases such as type-2 diabetes. Therefore, this issue should be investigated with more attention. The prevalence of obesity among children and adolescents in the United States reaches from 7% and 5% in 1980, to nearly 18% and 21% in 2012 [29]. In 2005, 9.7% (11.3% for boys, 8.0% for girls) of South Korean children and adolescents were obese; 19.0% were overweight or obese [30]. Based on the MONICA project (monitoring of cardiovascular diseases) in 1998, Iran was one of seven countries with a high prevalence of obesity among children [31]. In this study, increasing prevalence of obesity/overweight is totally detectible especially in stepwise studies [32-34]. The national maximum prevalence of overweight and obesity were 13.5 and 11.9 in our study which are similar to the result of mentioned studies. The range of national report of the prevalence of overweight (adult: 27.0- 38.5, <18 y: 5.0-13.5) and obesity (adult: 12.6-25.9, <18 y: 3.2-11.9) was totally different with sub national overweigh (adult: 12.8-76.4, <18 y: 2.4-67.1) and obesity (adult: 2.4-35.4, <18 y: 0.6-27.7) reports, that he wide reported range in sub national studies can be related to the lower number of sample size.

Although the estimated prevalence of overweight and obesity are different in various studies around the world, but it is increasing in most of them as a common factor [23]. There were seen extensive variations in prevalence of obesity/overweight in different regions of Iran as well, which may relate to various cultural affiliations, and socioeconomic factors among different provinces. Iran is a multiethnic country, so the multiplicities in socio-cultural, environmental, and genetic factors, directly affect eating habits and lifestyle in every region. Climate variability as an environmental factor influences on dietary habits, mood, and activity level. The urbanization phenomenon is considered as an important factor underlying obesity which is noticeably increasing in Iran. It seems lack of appropriate job opportunities, and the desire to take advantage of urban amenities, encourage the rural population to migrate to big cities such as Tehran. Although consumption of fast foods and not home-made foods as an important causes of obesity are increasing in major cities, in rural regions consuming home-made foods are still common. So we are faced a big problem, a huge and growing urban population and following unhealthy lifestyles that surly affect the increasing prevalence of obesity in near future. Jafar TH in their study found that the prevalence of obesity in urban inhabitants in Pakistan was 2.5 greater of obesity in inhabitants rural [27]. The prevalence of obesity and its acceleration rate are more perceptible among big cities of Iran, especially provincial capital [35-40]. In the latest overweight and obesity prevalence in Tehran, which was conducted in 2013 among 20–84 aged, the prevalence of overweight and obesity was reported 34.1% (95% CI: 32.3-35.9), and 15.4% (95% CI: 14.0-16.8), respectively [41]. In a survey in ten provinces of china, the prevalence rates of overweight and obesity were 1.4%-7.6% and 0.6%-3.1% among children [42]. These rates substantially are higher among children in our study. Although most of national and Iranian studies were asserted that the prevalence of overweight/obesity are greater in urban areas [43-46], but there are some studies with inconsistent result, such a study in Turkey which was reported the higher prevalence of obesity in rural (17%) in comparison to urban areas (15.2%) [26]. Despite these differences, all the stepwise studies showed a noticeable increasing in obesity/overweight prevalence in last decade in all age and sex groups [32,33,47,48].

In our review most of the studies reported higher prevalence of overweight and obesity among women than men [48-52], that may be due to lower physical activity among women in some areas.

## Conclusion

This review were assessed all related studies which were reported the prevalence of obesity and overweight in normal population of Iran since 2005. There was seen noticeable increase in overweight and obesity prevalence among all ages and both sex of Iranian people as the worldwide growing repots. Due to the higher population of young people in Iran, Ignoring the complications, and Lack of effective preventive policy, burden of obesity in the near future will be problematic. So mothers’ education, particularly among house workers has an important role in managing dietary habits in their family. Their awareness about the side effects of obesity can be more efficient which not only improve their health condition, but also provide a healthy life style for their children as well.

Considering a lot of life- threatening complications following overweight/obesity, it is very important to have national organized educational and preventive programs. Mapping of obesity in Iranian children and adults, and meta-analysis based on geographic and climate areas is recommended.

## Additional files

Additional file 1: Table S1. Prevalence of obesity and overweight in Iranian adults [1,2,35-41,44,45,47-50,52-73,51,74-120]. Additional file 2: Table S2. Prevalence of overweight and Obesity in Iranian under-18 individuals [21,32-34,56,92,94,95,121-220]. Additional file 3: Table S3. A summary on the prevalence of overweight and obesity in Iran.
