# Supplementary material for: Prevalence of obesity and overweight in adults and children in Iran; a systematic review
Source: J Diabetes Metab Disord. 2014 Dec 23;13:121. doi: 10.1186/s40200-014-0121-2 (PMC4301060; doi:10.1186/s40200-014-0121-2)
Supplement: Additional file 1: Table S1. — Prevalence of obesity and overweight in Iranian adults [1,2,35-41,44,45,47-50,52-73,51,74-120]. [file 40200_2014_121_MOESM1_ESM.docx]

**Table S1**. Prevalence of obesity and overweight in Iranian adults.

| **Reference** | **Study level** | **Year of publication** | **Location-**  **U/R** | **Age Range (yr)** | **N**  **T/M/F**  **U/R** | | **Prevalence of overweight (%)** | **95 % CI** | **Prevalence of obesity (%)** | **95 % CI** | **Overweight/obesity** | **95 % CI** |
| --- | --- | --- | --- | --- | --- | --- | --- | --- | --- | --- | --- | --- |
| Azadbakht L et al(53) | Local | 2005 | Tehran-U | 20-70 | T  M  F | 9984  4164  5820 |  |  |  |  | 51.1  29.0  67.0 | 50.1-52.1  27.6-30.4  65.8-68.2 |
| Wickramasinghe P et al(54) | Local | 2005 | Tabriz- U | 14-20 | F | 1650 | 11.1 | 9.6-12.7 | 3.6 | 2.7-4.5 |  |  |
| Pourshams A et al(55) | Local | 2005 | Tehran- U | >18 | T  M  F | 1965  1465  494 | 47.0  50.8  35.6 | 44.6-49.0  48.2-53.4  31.4-40.0 | 23.9  22.2  28.8 | 21.9-25.8  20.1-24.5  24.8-32.9 |  |  |
| Vahidinia A et al (56) | Local | 2005 | Hamedan-R | 21-87 | T  M  F | 344  158  186 | 25.3  24.7  25.8 | 20.8-30.2  18.1-32.1  19.7-32.7 | 9.6  5.7  12.9 | 6.7-13.2  2.6-10.5  8.4-18.6 |  |  |
| Mostafavi H et al (57) | Local | 2005 | Shiraz- U | >18 | T  M  F | 3245  1228  2017 | 33.3  28.2  36.8 | 31.7-35.0  25.7-30.8  34.7-38.9 | 11.7  7.3  14.8 | 10.6-12.7  5.9-8.9  13.2-16.4 |  |  |
| Agheli N et al (58) | Local | 2005 | Rasht &Ghazvin-U | >30 | T | 1100 |  |  | 34.8 | 32.0-37.7 |  |  |
|  |  |  | Rasht |  | T  M  F | 550  285  265 |  |  | 35.4  19.4  52.8 | 31.4-39.6  14.8-24.4  46.7-59.0 |  |  |
|  |  |  | Ghazvin |  | T  M  F | 550  274  276 |  |  | 34.2  21.0  47.4 | 30.2-38.3  16.1-26.1  41.4-53.5 |  |  |
| Mozafari M et al(59) | Local | 2006 | Ilam- U | 15-49 | F | 420 | 48.6 | 43.7-53.5 | 22.4 | 18.5-26.7 |  |  |
| AlavinaeiniAet al(60) | Local | 2006 | Isfahan- U | >60 | T  M  F | 1694  731  963 | 39.0  39.7  38.4 | 36.6-41.3  36.1-43.3  35.3-41.6 | 21.6  12.0  28.9 | 19.7-23.6  9.6-14.5  26.0-31.8 |  |  |
| Hajian K et al (61) | Province | 2006 | Mazandaran- U | 20-70 | T  M  F | 3600  1800  1800 | 34.8  36.2  33.5 | 33.3-36.4  33.9-38.4  31.3-35.7 | 18.8  9.9  27.8 | 17.5-20.1  8.5-11.3  25.7-29.9 |  |  |
| Sarshar N et al(62) | Local | 2006 | Gonabad- U | 15-65 | F | 440 | 27.7 | 23.6-32.1 | 14.5 | 11.4-18.2 |  |  |
| Abdollahi A et al(63) | Province | 2006 | Golestan-U | 17-70 | T  M  F | 5000  2500  2500 | 38.6  35.0  42.3 | 37.2-40.0  33.1-36.9  40.3-44.2 | 25.5  20.3  30.7 | 24.3-26.7  18.7-21.9  28.9-32.5 |  |  |
| Bahrami H et al(64) | Province | 2006 | Golestan- U & R | 35-81 | T  M  F | 8998  3786  5212 | 62.2  17.0  36.7 | 61.2-63.2  15.8-18.2  35.4-38.0 | 28.0  38.0  33.1 | 27.0-28.9  36.4-39.6  31.8-34.4 |  |  |
| Dastgiri S et al(39) | Local | 2006 | Tabriz- U | ≥18 | T M F | 300 132 168 | 43.3 40.9 45.2 | 37.6-49.1 32.4-49.8 37.5-53.1 | 22.4 18.0 24.0 | 18.0-27.6 12.5-25.6 18.5-31.4 |  |  |
| Rezaeian M et al (65) | Local | 2007 | South East of Iran- U | ≥ 30 | TMF | 756 319 437 | 37.3 36.9 37.5 | 33.8-40.8 31.7-42.5 33.0-42.2 | 12.3 4.7 18.2 | 10.0-14.8 2.6-7.6 14.4-21.8 |  |  |
| Amani R et al(66) | Local | 2007 | Ahwaz-U | 18-40 | T | 637 | 34.4 | 30.7-38.2 | 18.3 | 15.3-21.4 |  |  |
| Siasi F et al (67) | Local | 2007 | Kerman-R | 20-45 | F | 370 | 19.7 | 15.8-24.1 | 15.9 | 12.4-20.1 |  |  |
| Hajian-Tilaki K et al (68) | Province | 2007 | Mazandaran-U | 20-70 | TMF | 360018001800 | 34.8 36.2 33.5 | 33.2-36.4 33.9-38.4 31.1-35.6 | 18.8 9.9 27.8 | 17.5-20.1 8.5-11.4 25.7-29.8 |  |  |
| Veghari G R et al (69) | Local | 2007 | Gorgan- R | 14-39 | F | 2838 | 28.2 | 26.5-29.8 | 16.5 | 15.1-17.9 |  |  |
| Janghorbani M et al (2) | National | 2007 | Iran-U | 15-64 | T M F | 89404 45082 44322 | 32.4 32.0 32.4 | 31.9-32.5 31.5-32.4 32.0-32.9 | 17.6 10.9 24.5 | 17.3-17.8 10.5-11.0 24.0-24.8 |  |  |
| Mazloomzadeh S et al(70) | Province |  | Zanjan U | 15-64 | TMF | 2492 1251 1241 | 30.7 30.4 30.9 | 28.9-32.5 27.9-33.1 28.4-33.6 | 15.0 8.9 21.3 | 13.7-16.5 7.3-10.6 19.0-23.6 |  |  |
| Zare N et al(71) | Local | 2007 | Zarrindasht-R | 17-47 | F | 920 | 30.0 | 27.0-33.0 | 14.0 | 11.8-16.4 |  |  |
| Hosseinpanah F et al (72) | Province | 2007 | Tehran-U | >20 | T M F | 4728 1961 2767 | 42.3 | 40.9-43.7 | 23.7 15.1 29.8 | 22.5-24.9 13.5-16.7 28.1-31.5 |  |  |
| Kelishadi R et al (73) | Local | 2008 | Isfahan-U | >18 | T MF | 3694 1924 1770 | 36.3 36.6 35.9 | 34.7-37.9 34.4-38.8 33.7-38.2 | 22.1 11.2 28.1 | 18.1-20.7 9.8-12.8 26.0-30.3 |  |  |
| Kelishadi R et al(51) | National | 2008 | 28 provinces-U & R | 15-64 | T  M  F  U  R | 89532  45113  44344  57866  31666 | 28.6 | 28.3-28.9 | 14.2 | 14.0-14.4 | 42.8  37.0  48.0  46.7  35.5 | 42.5-43.1  36.5-37.4  47.5-48.5  46.3-47.1  35.0-36.0 |
| Bakhshi E et al(74) | National –NHS* | 2008 | Iran- U & R | 20-69 | F  U  R | 14176  8957  5219 |  |  | 17.5  21.0  11.5 | 16.9-18.1  20.1-21.8  10.6-12.4 |  |  |
| Nemati A et al (75) | Local | 2008 | Ardebil- U & R | >50 | F  U  R | 924  386  538 | 40.6  44.6  37.9 | 37.4-43.8  39.5-49.7  33.8-42.1 |  |  |  |  |
| Esteghamati A et al (76) | Local | 2008 | Tehran-U | 25-75 | T | 2309 |  |  | 34.6 | 32.6-36.5 |  |  |
| Nabipour I et al(77) | Province- PHHS | 2008 | Bushehr-U | ≥ 25 | T  M  F | 3723  1746  1977 | 22.6  24.3  21.1 | 21.3-24.0  22.3-26.4  19.4-23.0 | 14.9  9.0  20.1 | 13.8-16.1  7.7-10.5  18.4-22.0 |  |  |
| AlikhaniSet al(49) | National | 2009 | 30 provinces-U & R | 25-64 | T  M  F | 70,981  35,148  35,833 |  |  | 19.3  11.9  27.2 | 18.9–19.7  11.6–12.4  26.7–27.8 | 54.7  47.3  62.8 | 54.2–55.3  46.6–48.0  62.1–63.4 |
| Asgari F et al(78) | National | 2009 | 28 provinces- R & U | 15-64 | T | 89000 | 28.6 | 28.3-28.9 | 14.8 | 14.6-15.0 |  |  |
| Azimi-nezhad M et al(79) | province | 2009 | Khorasan- U & R | 15-65 | T  M  F | 4928  2483  2445 |  |  | 13.7  7.8  19.7 | 12.8-14.7  6.8-8.9  18.1-21.3 |  |  |
| Barzin M et al(80) | province | 2009 | Tehran- U | 18-25 | F | 926 | 11.9 | 9.9-14.1 | 3.1 | 2.1-4.5 |  |  |
| Tohidi M et al(81)‬‬‬‬‬‬‬ | province | 2009 | Shiraz- U | 19-95 | T  M  F | 855  293  562 | 37.1  42.7  34.1 |  | 17.9  10.5  21.9 |  |  |  |
| Delavar A et al (50) | National | 2009 | 30 provinces - U & R | 25–64 | T  M  F | 3024  1431  1535 | 34.2  32.1  36.1 | 32.5-35.9  29.7-34.6  33.7-38.5 | 25.1  17.2  33.3 | 23.6-26.7  15.3-19.2  30.9-35.7 |  |  |
| Esmaeily H et al(82) | Province | 2009 | Khorasan- U & R | 15-65 | T  M  F | 4977  2481  2496 | 29.0  27.7  30.5 | 27.8-30.4  25.9-29.5  28.7-32.3 | 13.7  7.8  19.7 | 12.8-14.8  6.7-8.9  18.2-21.3 |  |  |
| Esteghamati A et al (83) | National | 2009 | Iran- U & R | 15-64 | T | 5287 | 36.3 | 34.6-38.1 | 22.3 | 20.2-24.5 |  |  |
| Harati, H et al (84) | Local | 2009 | Tehran- U | ≥ 20 | T | 3307 | 40.7 | 39.0-42.4 | 23.3 | 21.2-24.8 |  |  |
| Sharifi, F et al (85) | Local | 2009 | Zanjan- U | > 20 | T | 2941 | 36.3 | 34.6-38.1 | 14.8 |  |  |  |
| Rashidy-Pour A et al(44) | Province | 2009 | Semnan- U | 30-70 | T  M  F  U  R | 3799  1695  2104  2715  1084 | 40.7  43.6  38.3  42.2  36.6 | 39.1-42.2  41.2-46.0  36.2-40.4  40.3-44.1  33.7-39.6 | 26.3  14.5  35.9  27.9  22.3 | 24.9-27.7  12.9-16.3  33.8-38.0  26.2-29.6  20.8-26.0 |  |  |
| Nematy, M et al(86) | Province | 2009 | Khorasan- U | >60 | T  M  F | 1962  917  1045 | 28.9  26.5  31.2 | 26.2-30.2  23.0-28.8  27.5-33.1 | 11.7  7.3  15.5 | 9.9-12.8  5.5-8.9  12.9-17.3 |  |  |
| Mohammad K et al (87) | Local | 2009 | Tehran- U | 15-49 | F | 2859 | 28.4 | 26.7-30.1 | 16.4 | 15.1-17.8 |  |  |
| Hosseinpanah F et al (48) | province- TLGS | 2009 | Tehran- U | >20 | Phase I  T  M  F | 4402  1835  2567 | 67.5  60.6  72.4 | 66.1-68.8  58.3-62.8  70.6-74.1 | 24.9  15.8  31.5 | 23.7-26.2  14.2-17.5  29.7-33.3 |  |  |
|  |  |  |  |  | Phase II  T  M  F | 4402  1835  2567 | 74.1  66.9  79.4 | 72.9-75.4  64.7-69.1  77.8-80.9 | 29.7  18.6  37.7 | 28.4-31.1  16.8-20.4  35.8-39.6 |  |  |
|  |  |  |  |  | Phase III  T  M  F | 4402  1835  2567 | 76.4  70.1  80.9 | 75.1-77.6  67.9-72.1  79.3-82.4 | 31.2  21.0  38.6 | 29.9-32.6  19.1-22.9  36.7-40.5 |  |  |
| Abdollahi, A A et al(88) | province | 2010 | Golestan- U | 17-70 | T  M  F | 5000  2500  2500 | 38.6  42.3  35.0 | 37.2-40.0  40.3-44.2  33.1-36.9 | 25.5  20.3  30.7 | 24.3-26.7  18.7-21.9  28.9-32.5 |  |  |
| Ayatollahi S M et al(1) | Local | 2010 | Shiraz-U | 25-55 | T  M  F | 2282  1141  1141 |  |  | 16.5  10.5  22.5 | 15.0-18.0  8.8-12.4  20.0-25.0 | 56.8  49.7  63.9 | 54.7-58.8  46.7-52.6  61.0-66.7 |
| Ebrahimi M et al(89) | National | 2010 | Iran- U & R | 15-64 | T | 29972 |  |  |  |  | 45.0 | 44.4-45.6 |
| Esteghamati A et al(90) | National | 2010 | Iran- U & R | 25-64 | T | 21576 | 1999:32.2  2005:35.8  2007:36.3 | 31.6-32.8  35.1-36.4  35.6-36.9 | 1999:13.6  2005:19.6  2007:22.3 | 13.1-14.0  19.1-20.1  21.7-22.8 |  |  |
| Heshmat R et al(35) | National | 2010 | Tehran,Tabriz,Shiraz,Mashhad,Bushehr-U | 19-65 | T  M  F | 5724  2396  3328 | 38.5  39.0  38.2 | 37.2-39.8  37.0-41.0  36.5-39.9 | 19.7  12.3  25.6 | 18.7-20.8  11.0-13.7  24.1-27.1 |  |  |
| Rostambeigi N et al (52) | National | 2010 | Iran | 25-65 | T  M  F | 8259  3634  4625 |  |  | 25.9  17.0  34.0 | 24.9-26.8  16.0-19.0  32.0-36.0 |  |  |
| Veghari G et al(91) | Province | 2010 | Golestan- U & R | 25-65 | T | 1999 | 33.1 | 31.1-35.3 | 29.0 | 27.0-31.0 |  |  |
| Veghari, G et al(45) | Local | 2010 | Golestan- U & R | 15-65 | T  M  F  U  R | 2495  1247  1248  1161  1334 | 30.3  32.9  28.6  32.3  29.5 | 28.5-32.2  30.0-35.3  25.6-30.6  29.1-34.6  26.6-31.5 | 24.2  15.8  32.7  29.4  19.8 | 22.6-26.0  13.8-18.0  30.135.4  26.8-32.1  17.7-22.0 |  |  |
| Ahmadnia A et al(92) | Local | 2010 | Zanjan- U | >15 | T | 1821 | 32.8 | 30.6-35.0 | 12.5 | 11.0-14.1 |  |  |
| Najafi I et al (93) | Province | 2010 | Golestan-U | ≥ 18 | T | 3591 | 35.0 | 33.4-36.6 | 24.5 | 23.1-26.0 |  |  |
| Maddah M et al (94) | Local | 2010 | Rasht-U | Adult | T  M  F | 12251  6028  6223 | 43.1  43.4  42.8 | 42.2-44.0  42.1-44.6  41.5-44.3 | 18.7  12.2  25.1 | 18.1-19.4  11.3-13.0  24.026.2 |  |  |
| Maddah M et al(95) | province | 2010 | Guilan- U & R | 50 -83 | F  U  R | 731  440  291 | 33.9  35.9  33.1 | 30.5-37.5  31.4-40.6  25.7-36.6 | 40.1  46.2  31.1 | 36.5-43.7  41.4-50.9  25.7-36.6 |  |  |
| Paknahad Z et al(96) | Local | 2010 | Isfahan- R | 16-85 | T | 1493 | 21.7 | 19.6-23.9 | 6.2 | 5.0-7.6 |  |  |
| Delavar M.A et al(97) | Local | 2011 | Babol- U | 30-50 | F | 809 |  |  |  |  | 82.8 | 80.0-85.3 |
| Naghashpour M et al(98) | province | 2011 | Khozestan- U | >18 | T  M  F | 252  68  184 | 30.7  16.7  32.6 | 24.9-36.6 | 33.6  17.9  34.9 |  |  |  |
| Hatmi Z N et al(99) | Local | 2011 | Tehran-U | ≥ 18 | T | 28566 | 42.6 | 42.0-43.1 | 13.7 | 13.3-14.1 | 56.3 | 55.7-56.9 |
| Sepandi M et al(100) | Local | 2011 | Ahvaz-U | 30.7±10.2 | F | 899 | 39.4 | 36.1-42.6 | 13.2 | 11. 1-15.6 |  |  |
| Heydari S T et al(101) | Local | 2011 | Shiraz-U | 18-27 | T  M  F | 288  153  135 | 12.8  15.7  9.6 | 9.2-17.3  10.3-22.4  5.2-15.9 | 2.4  2.6  2.2 | 1.0-4.9  0.7-6.5  0.4-6.3 |  |  |
| Sadeghi-bazargani H et al (102) | Local | 2011 | Ardabil- U | 15-64 | T | 1000 | 33.3 | 30.4-36.3 | 25.6 | 22.9-28.4 | 58.9 | 55.8-62.0 |
| Navadeh S et al(103) | National | 2011 | 28 provinces | 15-65 | F | 33472 | 34.5 | 34.0-35.0 | 24.5 | 24.0-25.0 |  |  |
| Namayandeh S M et al(104) | Local | 2011 | Yazd-U | 20-74 | T  M  F | 2000  1000  1000 | 36.1  43.3  62.0 | 34.0-38.2  40.2-46.4  58.9-65.0 | 16.5  9.1  24.3 | 14.9-18.2  7.4-11.0  21.7-27.1 |  |  |
| Hosseini S R et al(105) | province | 2011 | Amirkola-U | > 60 | T  M  F | 988  593  395 | 38.7  33.7  46.1 | 35.6-41.8  29.9-37.7  41.1-51.1 | 5.4  3.5  8.1 | 4.0-6.9  2.2-5.4  5.6-11.2 |  |  |
| Faramarzi H et al(106) | province | 2011 | Fars- R | ≥30 | T  M  F | 447251  241344  205907 |  |  | 10.2  10.5  9.9 | 10.1-10.3  10.4-10.6  9.7-10.0 |  |  |
| Moghadasi M et al(107) | Local | 2011 | Shiraz- U | 14-16 | M | 808 | 10.9 | 8.8-13.2 | 6.4 | 4.8-8.3 |  |  |
| MohammadiN et al (108) | Local | 2011 | Hamedan- U | 26-30 | F | 400 | 33.7 | 29.1-38.6 | 15.7 | 12.3-19.7 |  |  |
| NooriRet al(36) | Local | 2012 | Shiraz- U | 19-80 | T  M  F | 727  354  373 | 40.3  39.5  36.1 | 36.7-44.0  34.4-44.8  36.0-46.2 | 11.4  13.5  9.3 | 9.2-13.9  10.2-17.6  6.6-12.8 |  |  |
| Veghari G et al (46) | Province | 2012 | Golestan- U & R | 15-65 | T  M  F  U  R | 6489  3245  3244  2703  3786 | 31.5  32.4  30.5  32.7  29.9 | 30.4-32.7  30.8-34.0  29.0-32.1  31.9-35.5  28.4-31.4 | 23.0  14.1  31.8  25.5  21.2 | 22.0-24.0  13.0-15.4  30.2-33.4  23.9-27.2  19.9-22.5 |  |  |
| Biglaria A et al(109) | National | 2012 | Iran- U & R | 20-65 | T | 25307 |  |  | 12.6 | 12.2-13.0 |  |  |
| Gharakhanlou R et al(110) | National | 2012 | 7 big cities- U | 15-74 | T  M  F | 2179  991  1188 | 36.7  39.4  34.4 | 34.6-38.7  36.3-42.5  31.7-37.2 | 14.8  10.2  18.6 | 13.3-16.3  8.4-12.2  16.4-20.9 |  |  |
| Zarei S et al (111) | Local | 2012 | Jahrom-U | 20-85 | T  M  F | 1593  719  874 |  |  |  |  | 35.3  32.7  37.3 | 32.9-37.6  29.2-36.1  34.1-40.5 |
| ShiraniS et al(37) | Local | 2012 | Isfahan- U | 15-65 | T | 1000 |  |  |  |  | 60.8 | 57.7-63.9 |
| Najafipour H et al(112) | Local | 2012 | Kerman- U | 15-75 | T  M  F | 5900  2662  3238 | 42.0 | 40.7-43.3 | 12.0  9.2  16.8 | 11.2-12.8  8.5-10.0  16.1-17.6 |  |  |
| Poorolajal J et al(113) | Local | 2012 | Hamadan- U | 15-64 | T  M  F | 6500  3250  3250 | 32.1  28.6  35.7 | 30.9-33.2  27.0-30.2  34.0-37.4 | 15.1  8.0  22.3 | 14.2-16.0  7.1-9.0  20.9-23.8 |  |  |
| Jafari E et al(114) | province | 2012 | Golestan | 40-75 | T | 50045 | 33.9 | 33.5-34.3 | 25.4 | 25.0-25.8 |  |  |
| Veghari G et al (115) | Province | 2013 | Golestan- U | 15-65 | T  M  F  U  R | 2452  1224  1228  1141  1311 | 30.9  32.9  28.9  32.3  29.7 | 29.1-32.9  30.3-35.6  26.4-31.5  29.6-35.1  27.2-32.2 | 24.0  15.5  32.5  29.1  19.7 | 22.3-25.7  13.5-17.6  29.9-35.1  26.4-31.7  17.5-21.9 |  |  |
| Veghari G et al(116) | province | 2013 | Golestan- U & R | 15-65 | T  M  F | 2994  1499  1495 | 31.7  33.0  30.4 | 30.0-33.3  30.5-35.3  28.0-32.7 | 22.8  13.3  32.3 | 21.2-24.3  11.6-15.1  29.1-34.7 |  |  |
| HesarKoushki M et al(117) | Local | 2013 | Neyshabour-U | 30-50 | F | 381 | 45.5 | 40.3-50.5 | 30.4 | 25.9-35.3 |  |  |
| Moghimi-Dehkordi B et al (41) | Local | 2013 | Tehran- U | 20-84 | T | 2708 | 34.1 | 32.3-35.9 | 15.4 | 14.0-16.8 |  |  |
| Sarrafzadegan N et al(47) | Province-IHHP | 2013 | Isfahan-U | [2001:  38.9 ± 14.9 2007:  38.8 ± 15.6] | T  F  M | 6339  3119  3220 |  |  |  |  | 2001:55.4  2007: 60.0  2001: 37.9  2007: 40.8 | 53.6-57.1  58.2-61.7  36.2-39.6  39.1-42.5 |
| Rashidi H et al(118) | Province | 2013 | Ahvaz- U | 18-45 | F | 646 | 36.9 | 33.1-40.7 | 22.1 | 19.0-25.5 |  |  |
| Lankarani K B et al (38) | Local | 2013 | Shiraz- U | >18 | T  M  F | 819  340  479 | 39.3 | 35.9-42.7 | 15.6 | 13.1-18.3 | 17.9  20.9  15.9 | 15.4-20.7  16.7-25.6  12.7-19.4 |
| Kabir A et al (119) | Local | 2013 | Golestan- U | >50 | T  M  F | 1309  688  621 |  |  | 25.1  17.4  33.5 | 22.8-27.6  14.7-20.5  29.8-37.3 |  |  |
| Yarahmadi S et al(40) | National | 2013 | 6 provinces' capital cities-U | ≥ 30 | T  M  F | 439406  263644  175762 | 27.0  14.4  30.6 | 26.8-27.1  14.2-14.5  30.3-30.8 | 20.0  26.0  11.0 | 19.8-20.1  25.8-26.1  10.8-11.1 |  |  |
| Jahangiri H et al (120) | Province | 2013 | Tehran-U | 50-75 | T  M  F | 4380  2180  2200 |  |  | 11.2  6.5  14.6 | 10.3-12.2  4.6-6.5  15.3-18.5 |  |  |

**Legend: n:** number of study population, **CI:** confidence interval, **T:** total, **M:** male, **F:** female, **U:** Urban, **R:** Rural.

NHS, National Health Survey, TGLS: Tehran Lipid and Glucose Survey; PHHS, Persian Gulf Healthy Heart Study; NHS, National Health Survey; IHHP, the Isfahan Healthy Heart Program.

*Age in mean ± standard Deviation
