# Supplementary material for: Prevalence of obesity and overweight in adults and children in Iran; a systematic review
Source: J Diabetes Metab Disord. 2014 Dec 23;13:121. doi: 10.1186/s40200-014-0121-2 (PMC4301060; doi:10.1186/s40200-014-0121-2)
Supplement: Additional file 2: Table S2. — Prevalence of overweight and Obesity in Iranian under-18 individuals [21,32-34,56,92,94,95,121-220]. [file 40200_2014_121_MOESM2_ESM.docx]

**Table S2**. Prevalence of overweight and Obesity in Iranian under-18 individuals.

| **Reference** | **Study Level** | **Year** | **Location- U/R** | **Age (yr)** | **Standard criteria** | **N- T/M/F- U/R*** |  | **Prevalence of overweight (%)** | **95 % CI** | **Prevalence of obesity (%)** | **95 % CI** | **Overweight/obesity** | **95 % CI** |
| --- | --- | --- | --- | --- | --- | --- | --- | --- | --- | --- | --- | --- | --- |
|  |  |  |  |  |  |  |  |  |  |  |  |  |  |
| Valizade M et al(121) | Local | 2005 | Zanjan-U | 12-15 | WHO | F | 1010 |  |  | 3.8 | 2.7-5.1 |  |  |
| Sotoudeh G et al(122) | Local | 2005 | Islamshahr- U &R | 10-18 | CDC | F | 278 | 5.4 | 3.0-8.7 |  |  |  |  |
| Mostafavi H et al(123) | Local | 2005 | Shiraz-U | 13-18 | WHO | T | 803 | 11.3 | 9.2-13.7 | 2.9 | 1.9-4.4 |  |  |
|  |  |  |  |  |  | M | 377 | 8.2 | 5.6-11.5 | 2.6 | 1.3-4.8 |  |  |
|  |  |  |  |  |  | F | 426 | 14.0 | 10.9-17.7 | 3.2 | 1.8-5.4 |  |  |
| Soheilifar J et al(124) | Local | 2005 | Hamedan-U | 7-11 | WHO | T | 1390 | 6.4 | 5.2-7.8 | 5.7 | 4.6-7.1 |  |  |
|  |  |  |  |  |  | M | 691 | 5. 8 | 4.2-7.8 | 5.8 | 4.2-7.8 |  |  |
|  |  |  |  |  |  | F | 699 | 7.0 | 5.2-9.2 | 5.7 | 3.0-5.6 |  |  |
| Nader F et al(125) | Local | 2005 | Shiraz-U | 7-11 | WHO | T | 1300 |  |  | 2.9 | 2.1-4.0 |  |  |
|  |  |  |  |  |  | M | 674 |  |  | 1.6 | 0.8-2.9 |  |  |
|  |  |  |  |  |  | F | 626 |  |  | 4.3 | 2.9-6.2 |  |  |
| Bazhan M et al(126) | Local | 2005 | Lahijan-U | 14-17 | WHO | F | 400 | 14.8 | 11.4-18.6 | 5.3 | 3.3-7.9 |  |  |
| Jazayeri S M H M(127) | Local | 2005 | Tehran-U | 6-10 |  | T | 3104 | 21.1 | 19.7-22.6 | 8.0 | 7.1-9.1 |  |  |
|  |  |  |  |  | CDC | M | 1382 | 21.3 | 19.1-23.5 | 6.7 | 5.4-8.1 |  |  |
|  |  |  |  |  |  | F | 1722 | 21.0 | 19.1-23.0 | 9.2 | 7.8-10.6 |  |  |
|  |  |  |  |  | IOTF | T |  | 17.8 | 16.5-19.2 | 2.1 | 1.6-2.7 |  |  |
|  |  |  |  |  |  | M |  | 16.1 | 14.2-18.1 | 2.8 | 2.0-3.8 |  |  |
|  |  |  |  |  |  | F |  | 19.3 | 17.4-21.2 | 1.6 | 1.0-2.3 |  |  |
| Khaji A et al(128) | Local | 2006 | Tehran-U | 11 | WHO | T | 2766 | 10.7 | 9.6-11.9 | 6.3 | 5.4-7.3 |  |  |
|  |  |  |  |  |  | M | 1159 | 10.7 | 9.0-12.6 | 6.9 | 5.5-8.5 |  |  |
|  |  |  |  |  |  | F | 1607 | 12.8 | 11.2-14.5 | 7.1 | 5.6-8.8 |  |  |
| Montazeryfard F et al(129) | Local | 2006 | Zahedan-U | 11-14 | WHO | F | 687 | 8.7 | 6.7-11.1 | 1.7 | 0.9-3.0 |  |  |
| Vahidinia A et al(56) | Local | 2006 | Hamedan- R | 2-20 | CDC | T | 270 | 6.7 | 4.0-10.3 | 5.2 | 2.9-8.5 |  |  |
|  |  |  |  |  |  | M | 151 | 7.9 | 4.2-13.5 | 5.3 | 2.3-10.2 |  |  |
|  |  |  |  |  |  | F | 119 | 5.0 | 1.9-10.6 | 5.0 | 1.9-10.6 |  |  |
| Moayeri H et al(130) | Local | 2006 | Tehran-U | 11-17 | CDC | T | 2880 | 17.9 | 16.5-19.3 | 7.1 | 6.2-8.1 |  |  |
|  |  |  |  |  |  | M | 1180 | 21.1 | 18.8-23.5 | 7.8 | 6.3-9.5 |  |  |
|  |  |  |  |  |  | F | 1700 | 14.7 | 13.0-16.5 | 6.4 | 5.3-7.7 |  |  |
| Abtahi M et al(131) | National | 2007 | 28 different provinces-U | <5 | CDC | T | 2505 |  |  | 5.2 | 4.4-6.2 |  |  |
|  |  |  |  |  |  | M | 1235 |  |  | 4.5 | 3.4-5.7 |  |  |
|  |  |  |  |  |  | F | 1292 |  |  | 5.9 | 4.7-7.3 |  |  |
|  |  |  |  |  |  | U | 1436 |  |  | 5.4 | 4.2- 6.6 |  |  |
|  |  |  |  |  |  | R | 1108 |  |  | 3.4 | 2.4-4.7 |  |  |
| Amini M et al(132) | Local | 2007 | Tehran-U | 10-15 | CDC | T | 398 | 16.1 | 1.6-20.1 | 10.0 | 7.1-13.1 |  |  |
|  |  |  |  |  |  | M | 199 | 13.6 | 9.1-19.1 | 13.1 | 8.7-18.5 |  |  |
|  |  |  |  |  |  | F | 199 | 18.6 | 13.4-24.7 | 6.5 | 3.5-10.9 |  |  |
| Hajifaraji M et al(133) | Local | 2007 | Tehran-U | 12-15 | CDC | T | 780 | 12.0 | 9.8-14.5 | 13.0 | 10.7-15.5 |  |  |
| Maddah M(134) | Local | 2007 | Rasht- U | 14-17 | IOTF | F | 1054 | 21.9 | 19.4-24.5 | 5.3 | 4.1-6.9 |  |  |
| Sadeghi M et al(135) | Local | 2007 | Isfahan- U | 6-11 | WHO | T | 633 | 67.1 | 63.3-70.8 |  |  |  |  |
|  |  |  |  |  |  | M | 317 | 70.0 | 64.6-75.0 |  |  |  |  |
|  |  |  |  |  |  | F | 316 | 64.2 | 58.7-69.5 |  |  |  |  |
| Mozaffari H et al(136) | Local | 2007 | Tehran- U | 7-12 |  | T | 1800 | 13.3 | 11.7-14.9 | 7.7 | 6.4-8.9 |  |  |
| Gholamreza V et al(137) | Local | 2007 | North of Iran- U & R | 6 | WHO | T | 3154 | 17.4 | 16.1-18.8 | 4.7 | 4.0-5.5 |  |  |
| Ayatollahi S M et al(138) | Local | 2007 | Shiraz-U | 6-11 | CDC | T | 2397 |  |  |  | 4.6 | 3.8-5.5 |  |
|  |  |  |  |  |  | M | 1268 |  |  |  | 3.3 | 2.4-4.4 |  |
|  |  |  |  |  |  | F | 1129 |  |  |  | 6.1 | 4.8-7.7 |  |
| Dorosti AR et al(139) | Local | 2008 | Yazd-U | 6-11 | WHO | T | 3245 |  |  | 13.3 | 12.2-14.5 |  |  |
| Khabazkhoob M et al(140) | Local | 2008 | Dezfoul-U & R | 7-18 | NCHS | T | 5508 |  |  | 2.6 | 1.7-3.4 |  |  |
|  |  |  |  |  |  | M | 2429 |  |  | 2.9 | 1.8-4.0 |  |  |
|  |  |  |  |  |  | F | 2079 |  |  | 2.3 | 1.3-3.4 |  |  |
|  |  |  |  |  |  | U | 3698 |  |  | 3.0 | 2.0-4.0 |  |  |
|  |  |  |  |  |  | R | 1810 |  |  | 1.8 | 1.0-2.6 |  |  |
| Taheri F et al(141) | Local | 2008 | Birjand-U | 15-18 | CDC | T | 2230 | 6.1 | 5.1-7.2 | 2.3 | 1.7-3.0 |  |  |
|  |  |  |  |  |  | M | 1115 | 5.0 | 3.8-6.5 | 2.8 | 1.9-3.9 |  |  |
|  |  |  |  |  |  | F | 1115 | 7.1 | 5.6-8.7 | 1.8 | 1.1-2.7 |  |  |
| Golestan M et al(142) | Local | 2008 | Yazd-U | 11-13 | WHO | T | 794 | 12.9 | 10.6-15.4 | 6.5 | 4.9-8.5 |  |  |
|  |  |  |  |  |  | M | 395 | 17.0 | 13.4-21.0 | 8.8 | 6.2-12.1 |  |  |
|  |  |  |  |  |  | F | 399 | 8.8 | 6.2-12.0 | 4.3 | 2.5-6.7 |  |  |
| Kelishadi R et al(143) | National-CASPIANI | 2008 | 23 provinces | 6-11 | CDC | T | 21111 | 8.8 | 8.4-9.2 | 4.5 | 4.2-4.8 |  |  |
|  |  |  |  |  | IOTF |  |  | 11.3 | 10.9-11.7 | 2.9 | 2.7-3.1 |  |  |
|  |  |  |  |  | WHO |  |  | 10.1 | 9.7-10.5 | 4.8 | 4.5-5.1 |  |  |
| Dorosty AR et al(144) | Local | 2008 | Neishabour-U | 6-12 | CDC | T | 1471 |  |  | 4.6 | 6.5-10.4 |  |  |
|  |  |  |  |  | IOTF |  |  |  |  | 7.3 | 13.8-19.7 |  |  |
| Hajian K et al(145) | Local | 2008 | Babol-U | 7-12 | CDC | T | 1000 | 12.3 | 10.3-14.5 | 8.5 | 4.4-7.4 |  |  |
|  |  |  |  |  |  | M | 400 | 12.5 | 9.4-16.1 | 8.8 | 6.2-11.9 |  |  |
|  |  |  |  |  |  | F | 600 | 12.2 | 9.6-15.0 | 3.8 | 2.4-5.7 |  |  |
| Bidad K et al(146) | Local | 2008 | Tehran-U | 11-17 | CDC | F | 400 | 14.6 | 11.2-18.3 | 6.7 | 4.9-10.2 |  |  |
| Alavian SM et al(147) | Local | 2009 | Tehran-U | 7-18 | CDC | T | 966 | 19.6 | 17.1-22.2 | 7.9 | 6.2-9.7 |  |  |
|  |  |  |  |  |  | M | 433 | 14.9 | 11.8-18.7 | 8.8 | 6.3-11.8 |  |  |
|  |  |  |  |  |  | F | 533 | 23.2 | 19.7-27.1 | 7.1 | 5.1-9.6 |  |  |
| Houshiar Rad A et al(148) | National | 2009 | 28 province-U & R | <5 | WHO | T | 2562 |  |  |  |  | 5.2 | 4.4-6.1 |
|  |  |  |  |  |  | M | 1235 |  |  |  |  | 4.5 | 3.4-5.8 |
|  |  |  |  |  |  | F | 1292 |  |  |  |  | 5.9 | 4.7-7.4 |
|  |  |  |  |  |  | U | 1436 |  |  |  |  | 5.4 | 4.2-6.6 |
|  |  |  |  |  |  | R | 1108 |  |  |  |  | 5.4 | 4.1-6.9 |
| Eftekhari M et al(149) | Local | 2009 | Lar-U | 13-20 | CDC | F | 431 | 9.5 | 6.9-12.7 |  |  |  |  |
| Akhavan- Karbasi S et al(150) | Local | 2009 | Yazd-U | 6 | WHO | T | 400 | 4.2 | 2.5-6.7 | 3.8 | 2.1-6.1 |  |  |
|  |  |  |  |  |  | M | 200 | 5.0 | 2.4-9.0 | 5.5 | 2.8-9.6 |  |  |
|  |  |  |  |  |  | F | 200 | 3.5 | 1.4-7.1 | 2.0 | 0.5-5.0 |  |  |
| Ataei N et al(151) | Local | 2009 | Tehran- U | 1-6 | CDC | T | 3186 | 7.2 | 6.3-8.1 | 12.2 | 11.1-13.4 |  |  |
|  |  |  |  |  |  | M | 1595 | 6.9 | 5.7-8.2 | 13.9 | 12.2-15.7 |  |  |
|  |  |  |  |  |  | F | 1591 | 7.5 | 6.2-8.9 | 10.5 | 9.0-12.1 |  |  |
| Maddah M et al(152) | Local | 2009 | Gilan- U & R | 15-18 | IOTF | F | 2302 | 36.6 | 34.6-38.6 | 9.3 | 8.1-10.5 |  |  |
|  |  |  |  |  |  | U | 1106 | 18.0 | 16.9-23.2 | 4.1 | 3.8 -4.5 |  |  |
|  |  |  |  |  |  | R | 1196 | 18.6 | 16.1-25.2 | 5.2 | 4.6-5.7 |  |  |
| Taheri F et al(153) | Local | 2009 | Birjand- U | 7-18 | CDC | T | 6093 | 4.7 | 4.2-5.3 | 1.8 | 1.5-2.2 |  |  |
|  |  |  |  |  |  | M | 3098 | 3.7 | 3.1-4.4 | 2.1 | 1.6-2.7 |  |  |
|  |  |  |  |  |  | F | 2995 | 5.8 | 5.0-6.7 | 1.5 | 1.1-2.0 |  |  |
| Veghari G et al(154) | Local | 2009 | Gorgan-R | 2-5 | WHO | T | 1446 | 15.3 | 13.5-17.3 | 27.7 | 25.4-30.0 |  |  |
|  |  |  |  |  |  | M | 713 | 14.9 | 12.3-17.7 | 28.9 | 25.6-32.4 |  |  |
|  |  |  |  |  |  | F | 733 | 15.9 | 13.2-18.7 | 26.5 | 23.3-29.8 |  |  |
| Salem Z et al(155) | Local | 2009 | Rafsanjan- U | 11-18 | CDC | F | 1221 | 11.2 | 9.4-12.9 | 2.4 | 1.5-3.3 |  |  |
| Faghih S et al(156) | Local | 2009 | Ahwaz-U | 14-18 | CDC | F |  |  |  |  |  |  |  |
|  |  |  |  |  |  | 1997 | 398 | 1.5 | 0.5-3.2 | 6.0 |  |  |  |
|  |  |  |  |  |  |  |  |  |  |  | 3.9-8.8 |  |  |
|  |  |  |  |  |  | 2006 | 420 | 5.4 | 3.5-8.1 | 13.5 | 10.4-17.2 |  |  |
| Maddah M et al(157) | Local | 2009 | Guilan- U & R | 14-17 | IOTF | F | 2090 | 32.4 | 30.4-34.5 |  |  | 22.9 | 21.0-24.7 |
|  |  |  |  |  |  | U | 1054 |  |  |  |  | 22.0 | 19.5-24.6 |
|  |  |  |  |  |  | R | 1036 |  |  |  |  | 23.8 | 21.2-26.4 |
| Maddah M et al(158) | Local | 2009 | Gilan- R | 14-17 | IOTF | F | 1036 | 18.6 | 16.3-21.1 | 5.2 | 3.9-6.7 |  |  |
| Fallahzadeh H et al(159) | Local | 2009 | Yazd-U | 11-13 | CDC | T | 800 | 13.0 | 10.7-15.5 |  |  |  |  |
|  |  |  |  |  |  | M | 400 | 17.5 | 13.9-21.6 |  |  |  |  |
|  |  |  |  |  |  | F | 400 | 8.5 | 5.9-11.7 |  |  |  |  |
| HaeriBehbahani B et al(160) | Local | 2009 | Sabzevar-U | 6-11 | CDC | T | 1800 | 7.9 | 6.7-9.2 | 4.8 | 3.8-5.8 |  |  |
|  |  |  |  |  |  | M | 960 | 8.4 | 6.7-10.2 | 6.0 | 4.5-7.6 |  |  |
|  |  |  |  |  |  | F | 840 | 7.4 | 5.6-9.2 | 3.5 | 2.2-4.7 |  |  |
| Montazerifar F et al(161) | Local | 2009 | Sistanva Baluchistan-U | 14-18 | CDC | F | 752 | 8.6 | 6.7-10.9 | 1.5 | 0.7-2.6 |  |  |
| Fesharakinia A et al(162) | Local | 2009 | south khorasan- U & R | 9-11 | CDC | T | 954 | 3.4 | 2.3-4.8 | 1.8 | 1.0-2.8 |  |  |
| Rahmanpour H et al(163) | Province | 2009 | Zanjan-U | 14-18 | WHO | F | 1882 | 8.9 | 7.7-10.3 | 1.4 | 0.9-2.1 |  |  |
| Mirhosseini N Z et al(164) | Local | 2009 | Mashhad-U | 15-17 | WHO | F | 622 | 14.6 | 11.9-17.6 | 3.4 | 2.1-5.1 |  |  |
| Rafraf M et al(165) | Local | 2010 | Tabriz- U | 15-18 | CDC | F | 985 | 16.4 | 14.2-18.9 | 2.8 | 1.9-4.1 |  |  |
| Ahmadnia A et al(92) | Local | 2010 | Zanjan-U | > 15 | CDC | T | 1821 | 32.8 | 30.6-35.0 |  |  | 12.5 | 11.0-14.1 |
| Ahmadi S et al(166) | Local | 2010 | Sanandaj Kurdistan-U | 14-18 | WHO | T | 694 | 11.2 | 12.0-13.8 | 3.2 | 2.0-4.8 | 14.4 | 11.9-17.2 |
|  |  |  |  |  |  | M | 340 | 12.9 | 9.6-17.0 | 5.3 | 3.2-8.2 | 18.2 | 14.3-22.7 |
|  |  |  |  |  |  | F | 354 | 9.6 | 6.7-13.1 | 1.1 | 0.3-2.8 | 10.7 | 7.7-14.4 |
| Mortazavi  Z et al(167) | Local | 2010 | Zahedan-U | 7-18 | CDC | M | 1278 | 5.6 | 4.3-6.9 | 3.1 | 2.2-4.2 |  |  |
| Mazaheri M et al(168) | Local | 2010 | Isfahan-U | 14-18 | WHO | F | 384 | 9.1 | 6.4-12.4 | 1.0 | 0.2-2.6 |  |  |
| Ahmadi E et al(169) | Local | 2010 | Kerman-U | 7-11 | CDC | T | 1566 | 4.4 | 3.4-5.5 | 0.6 | 0.3-1.1 |  |  |
|  |  |  |  |  |  | M | 770 | 4.3 | 3.0-6.0 | 1.0 | 0.4-2.0 |  |  |
|  |  |  |  |  |  | F | 796 | 4.5 | 3.2-6.2 | 0.1 | 0.0-0.6 |  |  |
| Hosseini M et al(170) | National | 2010 | Iran- U | 7-18 | CDC | T | 14865 | 10.4 | 10.2-11.6 | 5.6 | 5.2-5.9 |  |  |
|  |  |  |  |  |  | M | 7635 | 10.9 | 10.2-11.6 | 7.4 | 6.8-8.0 |  |  |
|  |  |  |  |  |  | F | 7230 | 9.9 | 9.2-10.6 | 3.6 | 3.2-4.0 |  |  |
| Maddah M et al(95) | Local | 2010 | Rasht- U | 12-17 | IOTF | F | 2577 | 18.6 | 17.1-20.1 | 5.9 | 5.0-6.9 |  |  |
| Salem Z et al(171) | Local | 2010 | Rafsanjan-U | 7-11 | CDC | T | 1275 | 9.4 | 7.9-10.9 |  |  |  |  |
|  |  |  |  |  |  | M | 500 | 10.2 | 7.7-13.2 |  |  |  |  |
|  |  |  |  |  |  | F | 775 | 8.9 | 7.0-11.1 |  |  |  |  |
| Maddah M et al(94) | Local | 2010 | Rasht- U | 6-11 | IOTF | T | 6635 | 13.1 | 12.3-13.9 | 5.4 | 4.9-6.0 |  |  |
|  |  |  |  |  |  | M | 3551 | 11.5 | 10.4-12.6 | 5.0 | 4.3-5.7 |  |  |
|  |  |  |  |  |  | F | 3084 | 15.0 | 13.7-16.3 | 5.9 | 5.1-6.8 |  |  |
| Gargari B et al(172) | Local | 2010 | Tabriz-U | 14-18 | CDC | F | 1887 | 3.6 | 2.8-4.5 |  |  |  |  |
| Mohammadian S et al(173) | Local | 2010 | Gorgan-U | 11-13 | WHO | F | 844 | 14.7 | 12.4-17.3 | 6.3 | 4.7-8.1 |  |  |
| MaddahM et al(174) | Local | 2010 | Rasht- U | 6-17 | IOTF | T | 8608 | 14.0 | 13.3-14.7 |  |  |  |  |
|  |  |  |  |  |  | M |  |  |  |  |  |  |  |
|  |  |  |  |  |  | F |  |  |  |  |  |  |  |
| Vafa M et al(175) | Local | 2010 | Tehran-U | 7-11 | WHO | T | 513 | 8.0 | 5.8-10.7 | 11.7 | 9.0-14.8 |  |  |
| Dahri M et al(176) | Local | 2010 | Mashhad-U | 11-15 | WHO | F | 1300 | 11.5 | 9.8-13.3 | 10.3 | 8.7-12.1 |  |  |
| Maddah M et al(177) | Local | 2010 | Zahedan-U | 6-10 | CDC | T | 1079 |  |  |  |  | 9.6 | 7.9-11.5 |
|  |  |  |  |  |  | M | 500 |  |  |  |  | 8.9 | 6.4-11.6 |
|  |  |  |  |  |  | F | 579 |  |  |  |  | 10.3 | 8.0-13.1 |
| Ziaoddini H et al(178) | National | 2010 | 31 provinces | 7 |  | T | 899 035 | 13.5 | 13.4-13.6 | 3.5 | 3.4-3.6 |  |  |
| Ahmadi S et al(179) | Local | 2010 | Sanandaj-U | 14-18 | CDC | T | 694 | 11.2 | 9.0-13.8 | 3.2 | 2.0-4.8 | 14.4 | 11.9-17.2 |
|  |  |  |  |  |  | M | 340 |  |  |  |  | 18.2 | 14.3-22.7 |
|  |  |  |  |  |  | F | 354 |  |  |  |  | 10.7 | 7.7-14.4 |
| Bazhan M et al(180) | Local | 2011 | Lahijan-U | 14-17 | CDC | F | 400 | 14.8 | 11.4-18.6 | 5.3 | 3.3-7.9 |  |  |
| Kakhak  S et al(181) | Local | 2011 | Sabzevar-U | 12-14 | CDC | M | 386 | 9.2 | 6.4-12.4 | 3.7 | 2.0-6.1 |  |  |
| Mohkam M et al(182) | Local | 2011 | Tehran-U | 7-11 | CDC | T | 425 | 12.0 | 9.1-15.5 |  |  |  |  |
| Mirzaeian S et al(183) | Local | 2011 | Najafabad-U | 15-18 | IOTF | F | 550 | 20.5 | 17.2-24.2 | 6.0 | 4.1-8.3 |  |  |
| Hajian-Tilaki K et al(184) | Local | 2011 | Babol-U | 7-12 | CDC | T | 1000 | 12.3 | 10.3-14.5 | 5.8 | 4.4-7.4 |  |  |
|  |  |  |  |  |  | M | 450 | 12.5 | 9.5-15.8 | 8.8 | 6.4-11.9 |  |  |
|  |  |  |  |  |  | F | 550 | 12.2 | 9.5-15.2 | 3.8 | 2.1-5.3 |  |  |
| Alvani S et al(185) | Local | 2011 | Kashan-U | 16-19 | CDC | T | 450 | 10.9 | 8.2-14.1 | 4.2 | 2.6-6.5 |  |  |
|  |  |  |  |  |  | M | 195 | 9.7 | 6.0-14.8 | 7.2 | 4.0-11.7 |  |  |
|  |  |  |  |  |  | F | 255 | 11.7 | 8.1-16.4 | 2.0 | 0.6-4.5 |  |  |
| Gaeini A et al(186) | Local | 2011 | Tehran-U | 3-6 | CDC | T | 756 | 10.0 | 8.0-12.4 | 4.6 | 3.2-6.4 |  |  |
|  |  |  |  |  |  | M | 378 | 9.8 | 7.0-13.2 | 4.8 | 2.8-7.4 |  |  |
|  |  |  |  |  |  | F | 378 | 10.3 | 7.4-13.8 | 4.5 | 2.6-7.1 |  |  |
| Veghari G et a(187) | Province | 2011 | Northern Iran- U & R | 7-11 | CDC | T | 7399 | 8.4 | 7.7-9.0 | 14.1 | 13.3-14.9 |  |  |
|  |  |  |  |  |  | M | 3934 | 8.7 | 7.8-9.6 | 15.3 | 14.2-16.5 |  |  |
|  |  |  |  |  |  | F | 3465 | 8.0 | 7.1-8.9 | 12.6 | 11.5-13.8 |  |  |
|  |  |  |  |  |  | U | 3664 | 8.4 | 7.5-9.3 | 16.8 | 15.6-18.1 |  |  |
|  |  |  |  |  |  | R | 3735 | 8.4 | 7.4-9.3 | 11.3 | 10.3-12.4 |  |  |
| Mirmohammadi S J et al(188) | National | 2011 | six ethnic groups | 7-18 | IOTF | T | 29988 | 9.3 | 8.9-9.5 | 3.2 | 3.0-3.4 |  |  |
|  |  |  |  |  |  | M | 14924 | 10.5 | 10.0-11.0 | 3.7 | 3.4-4.0 |  |  |
|  |  |  |  |  |  | F | 14964 | 8.1 | 7.7-8.5 | 2.7 | 2.4-3.0 |  |  |
|  |  |  |  |  | WHO |  |  | 12.7 | 12.2-13.0 | 10.4 | 10.0-10.7 |  |  |
|  |  |  |  |  |  |  |  | 11.8 | 11.3-12.3 | 12.1 | 11.6-12.6 |  |  |
|  |  |  |  |  |  |  |  | 13.5 | 12.9-14.0 | 8.9 | 8.4-9.4 |  |  |
| Taheri F et al(189) | Local | 2011 | Birjand-U | 11-15 | CDC | T | 2105 | 5.2 | 4.3-6.2 | 2.1 | 1.5-2.8 |  |  |
|  |  |  |  |  |  | M | 1067 | 3.9 | 2.8-5.3 | 2.8 | 1.9-4.0 |  |  |
|  |  |  |  |  |  | F | 1038 | 6.5 | 5.0-8.1 | 1.5 | 0.8-2.5 |  |  |
| Zareie et al(190) | Local | 2011 | Sabzevar-U | 12-14 | CDC | M | 650 | 9.8 | 7.7-12.4 | 7.1 | 5.2-9.3 |  |  |
| Kajbaf T Z et al(191) | province | 2011 | Ahvaz-U | 7-11 | CDC | T | 903 | 9.5 | 7.7-11.6 | 6.9 | 5.3-8.7 |  |  |
|  |  |  |  |  |  | M | 308 | 11.0 | 7.8-15.1 | 9.4 | 6.4-13.2 |  |  |
|  |  |  |  |  |  | F | 595 | 8.7 | 6.6-11.3 | 5.5 | 3.8-7.7 |  |  |
| Azarbayejani M et al(192) | Local | 2011 | Tehran-U | 7-11 | CDC | F | 488 | 18.8 | 15.5-22.6 | 11.4 | 11.1-17.5 |  |  |
| Moghadasi M et al(193) | Local | 2011 | Shiraz-U | 14-16 | CDC | M | 808 | 10.9 | 8.8-13.2 | 6.4 | 4.8-8.3 |  |  |
| AsadiNoghabi F et al(194) | Local | 2011 | Band Abbas-U | 7-11 |  | T | 1350 | 11.4 | 9.7-13.2 | 8.5 | 7.1-10.1 |  |  |
|  |  |  |  |  | CDC | M | 661 | 12.8 | 10.4-15.6 | 10.9 | 8.6-13.5 |  |  |
|  |  |  |  |  |  | F | 689 | 10.0 | 7.9-12.5 | 6.2 | 4.5-8.3 |  |  |
| Mirzaei M et al(195) | Local | 2011 | Yazd-U | 6-7 | WHO | T | 2768 | 6.3 | 5.4-7.2 | 2.4 | 1.8-3.0 |  |  |
|  |  |  |  |  |  | M | 1507 | 5.9 | 4.8-7.2 | 2.6 | 1.8-3.5 |  |  |
|  |  |  |  |  |  | F | 1261 | 6.7 | 5.3-8.2 | 2.2 | 1.5-3.2 |  |  |
| Amanolahi A et al(196) | Local | 2011 | Tehran-U | 7-11 | CDC | F | 1040 | 16.5 | 14.2-18.8 | 8.6 | 7.0-10.5 |  |  |
|  |  |  |  |  | WHO |  |  | 21.8 | 19.3-24.5 | 8.3 | 6.7-10.1 |  |  |
| Noohjah S et al(197) | Local | 2011 | Ahvaz-U | 2-5 | WHO | T | 1035 | 15.0 | 12.6-17.0 | 7.3 | 5.7-9.0 |  |  |
|  |  |  |  |  |  | M | 512 | 16.7 | 16.5-20.1 | 9.0 | 6.6-11.8 |  |  |
|  |  |  |  |  |  | F | 523 | 13.3 | 10.6-16.6 | 5.6 | 3.7-7.9 |  |  |
| Ghergherechi R et al(198) | Local | 2011 | Tabriz-U | 7-17 | WHO | F | 1000 | 12.8 | 10.8-15.0 | 9.5 | 7.7-11.5 |  |  |
| Motlagh M E et al(34) | National | 2011 | 30 provinces | 6 | CDC | T 2007 | 862433 | 12.8 | 12.7-12.9 | 3.4 | 3.3-3.4 |  |  |
|  |  |  |  |  |  | T 2008 | 782244 | 13.5 | 13.4-13.6 | 3.5 | 3.4-3.5 |  |  |
|  |  |  |  |  |  | T 2009 | 955388 | 10.9 | 10.8-11.0 | 3.4 | 3.3-3.4 |  |  |
| Mozaffari -hosraviH.et al(199) | Local | 2011 | Yazd-U | 14-18 | CDC | F | 1400 | 12.8 | 11.1-14.6 | 3.9 | 3.0-5.1 | 16.7 | 14.8-18.8 |
| Behzadnia S et al(200) | Local | 2011 | Sari-U | 7-12 | CDC | T | 653 | 27.1 | 23.7-30.7 | 12.0 | 9.5-14.7 |  |  |
| Ebrahimzadehkar B et al(201) | Local | 2011 | Golestan-U | 2-5 | WHO | T | 343 |  |  | 6.4 | 4.0-9.5 |  |  |
| Vafa M et al(202) | Local | 2012 | Tehran- U | 7 | WHO | T | 511 |  |  |  |  | 19.7 | 16.4-23.5 |
|  |  |  |  |  |  | M | 235 |  |  |  |  | 17.1 | 12.4-22.4 |
|  |  |  |  |  |  | F | 276 |  |  |  |  | 22.1 | 17.3-27.4 |
| MohamadpourKoldeh M et al(203) | Local | 2012 | Bushehr-U | 14-17 | CDC | F | 500 | 14.5 | 11.4-17.8 | 7.1 | 4.9-9.6 |  |  |
| Ebrahimzade B et al(204) | Local | 2012 | Bandar Turkmen-U | <5 | WHO | T | 616 |  |  | 6.0 | 4.2-8.2 |  |  |
| Mehrkash M et al(205) | Local | 2012 | northern Iran | 15-18 | CDC | T | 450 |  |  |  |  | 10.5 | 7.8-13.6 |
| Aazami M et al(206) | Local | 2012 | Kermanshah-U | 6-11 | CDC | T | 1400 | 13.4 | 11.6-15.2 | 10.9 | 9.3-12.6 |  |  |
|  |  |  |  |  |  | M | 756 | 12.6 | 10.3-15.1 | 13.4 | 11.0-16.0 |  |  |
|  |  |  |  |  |  | F | 644 | 14.3 | 11.7-17.2 | 8.1 | 6.1-10.4 |  |  |
| Barzin M et al(33) | Province-TGLS | 2012 | Tehran-U | 6-18 | CDC | Phase I |  |  |  |  |  |  |  |
|  |  |  |  |  |  | T | 3853 | 18.5 | 17.3-19.8 | 7.1 | 6.3-7.9 |  |  |
|  |  |  |  |  |  | M | 1858 | 19.7 | 17.9-21.6 | 8.3 | 7.1-9.6 |  |  |
|  |  |  |  |  |  | F | 1995 | 17.5 | 15.8-19.2 | 6.0 | 5.0-7.1 |  |  |
|  |  |  |  |  |  | Phase II |  |  |  |  |  |  |  |
|  |  |  |  |  |  | T | 3056 | 25.8 | 24.3-27.4 | 10.0 | 9.0-11.1 |  |  |
|  |  |  |  |  |  | M | 1415 | 28.3 | 25.9-30.7 | 11.9 | 10.2-13.7 |  |  |
|  |  |  |  |  |  | F | 1641 | 23.7 | 21.7-25.8 | 8.4 | 7.1-9.8 |  |  |
|  |  |  |  |  |  | Phase III |  |  |  |  |  |  |  |
|  |  |  |  |  |  | T | 3441 | 30.5 | 28.9-32.0 | 10.9 | 9.9-12.0 |  |  |
|  |  |  |  |  |  | M | 1592 | 34.3 | 32.0-36.7 | 13.8 | 12.2-15.6 |  |  |
|  |  |  |  |  |  | F | 1849 | 27.2 | 25.2-29.3 | 8.9 | 7.2-9.8 |  |  |
| Doustmohammadian A et al(207) | National | 2012 | Iran-U | 11-19 | CDC | T | 7908 | 5.0 | 4.5-5.5 | 3.3 | 2.9-3.7 |  |  |
|  |  |  |  |  |  | M | 4158 | 6.2 | 5.5-7.0 | 3.3 | 2.8-3.9 |  |  |
|  |  |  |  |  |  | F | 3750 | 8.7 | 7.8-9.6 | 3.3 | 2.7-3.9 |  |  |
| Taheri F et al(208) | Local | 2012 | Birjand-U | 2-5 | CDC | T | 500 | 10.6 | 8.0-13.6 | 7.6 | 5.4-10.3 |  |  |
|  |  |  |  |  |  | M | 261 | 9.6 | 6.3-13.8 | 9.6 | 6.3-13.8 |  |  |
|  |  |  |  |  |  | F | 239 | 11.7 | 7.9-16.5 | 6.3 | 3.5-10.1 |  |  |
| Kelishadi R et al(32) | National-IHHP | 2012 | Isfahan (reference area)- U | 11-18 | CDC | Phase I |  |  |  |  |  |  |  |
|  |  |  |  |  |  | T | 486 |  |  |  |  | 11.3 | 8.6-14.5 |
|  |  |  |  |  |  | M | 217 |  |  |  |  | 16.6 | 11.5-21.7 |
|  |  |  |  |  |  | F | 269 |  |  |  |  | 7.4 | 4.6-11.2 |
|  |  |  |  |  |  | Phase II |  |  |  |  |  |  |  |
|  |  |  |  |  |  | T | 515 |  |  |  |  | 17.7 | 14.5-21.2 |
|  |  |  |  |  |  | M | 259 |  |  |  |  | 21.2 | 16.4-26.7 |
|  |  |  |  |  |  | F | 256 |  |  |  |  | 14.1 | 10.0-18.9 |
| Hajian-Tilaki K et al(209) | Local | 2012 | Babol-U | 12-17 | CDC | T | 1200 | 15.1 | 13.1-17.2 | 8.3 | 6.8-10.0 |  |  |
|  |  |  |  |  |  | M | 600 | 15.3 | 12.5-18.4 | 10.2 | 7.9-12.9 |  |  |
|  |  |  |  |  |  | F | 600 | 14.9 | 12.1-17.9 | 6.5 | 4.7-8.8 |  |  |
| Khodaverdi F et al(210) | Local | 2012 | Tehran- U | 9-11 | CDC | T | 240 | 13.8 | 9.6-18.8 | 14.6 | 10.4-19.7 |  |  |
| Esmaeilzadeh S et al(211) | Local | 2012 | Ardabil-U | 7-11 | CDC | M | 766 | 14.1 | 11.7-16.8 | 4.2 | 2.9-5.8 |  |  |
| Zakeri M et al(212) | National- GSHS-WHO(CASPIAN II) | 2012 | All province- U | 10-18 | CDC | T | 8460 | 10.3 | 9.5-10.8 | 6.5 | 5.8-6.9 |  |  |
|  |  |  |  |  |  | M | 4524 | 10.0 | 9.0-10.7 | 7.5 | 6.6-8.2 |  |  |
|  |  |  |  |  |  | F | 3936 | 10.7 | 9.5-11.5 | 5.3 | 4.5-5.9 |  |  |
| Bahreini N et al(21) | Local | 2013 | Isfahan-U | 11-18 | IOTF | T | 3002 | 34.6 | 32.9-36.4 | 1.8 | 1.3-2.3 |  |  |
|  |  |  |  |  |  | M | 1377 | 29.6 | 27.2-32.1 | 2.1 | 1.5-3.1 |  |  |
|  |  |  |  |  |  | F | 1625 | 38.9 | 36.5-41.3 | 1.4 | 0.9-2.1 |  |  |
|  |  |  |  |  | CDC | T |  | 6.4 | 5.5-7.3 | 1.2 | 0.8-1.7 |  |  |
|  |  |  |  |  |  | M |  | 6.2 | 5.0-7.7 | 1.5 | 0.9-2.3 |  |  |
|  |  |  |  |  |  | F |  | 6.4 | 5.3-7.8 | 1.0 | 0.6-1.6 |  |  |
|  |  |  |  |  | WHO | T |  | 5.6 | 4.8-6.5 | 3.5 | 2.9-4.2 |  |  |
|  |  |  |  |  |  | M |  | 5.3 | 4.2-6.6 | 3.8 | 2.8-4.9 |  |  |
|  |  |  |  |  |  | F |  | 5.9 | 4.8-7.2 | 3.3 | 2.4-4.2 |  |  |
|  |  |  |  |  | National Iranian | T |  | 5.8 | 5.0-6.7 | 1.8 | 1.3-2.3 |  |  |
|  |  |  |  |  |  | M |  | 5.6 | 4.4-6.9 | 2.2 | 1.5-3.1 |  |  |
|  |  |  |  |  |  | F |  | 6.0 | 4.9-7.3 | 1.4 | 0.9-2.1 |  |  |
| Basiratnia M et al(213) | Province | 2013 | Fars –U | 11-17 | CDC |  | 2000 | 13.0 | 11.5-14.5 | 7.0 | 5.9-8.2 |  |  |
|  |  |  |  |  |  |  | 953 | 10.8 | 8.9-12.9 | 7.9 | 6.2-9.8 |  |  |
|  |  |  |  |  |  |  | 1047 | 15.0 | 12.9-17.3 | 6.2 | 4.8-7.8 |  |  |
| Ghavamzadeh S et al(214) | Local | 2013 | Urmia-U | 11-20 | CDC | T | 2498 |  |  |  |  | 14.1 | 12.7-15.5 |
| Hajian-Tilaki K et al(215) | Local | 2013 | Babol-U | 2-5 |  | T | 760 | 11.8 | 9.6-14.3 | 1.5 | 12.5-17.7 |  |  |
|  |  |  |  |  |  | M | 375 | 11.5 | 8.4-15.1 | 16.8 | 13.1-21.0 |  |  |
|  |  |  |  |  |  | F | 385 | 12.2 | 8.4-15.1 | 13.2 | 10.0-17.0 |  |  |
| Taheri F et al(216) | Local | 2013 | Birjand- U | 6-11 | CDC | T | 1541 | 9.6 | 8.0-11.2 | 9.2 | 7.8-10.8 |  |  |
|  |  |  |  |  |  | M | 690 | 11.0 | 8.8-13.6 | 10.9 | 8.6-13.4 |  |  |
|  |  |  |  |  |  | F | 851 | 8.3 | 6.7-10.5 | 7.9 | 6.1-9.9 |  |  |
| Salehi-Abargouei A et al(217) | Local | 2013 | Zabol- U | 11-15 | IOTF | T | 837 | 10.8 | 8.7-13.0 | 2.2 | 1.3-3.4 |  |  |
|  |  |  |  |  | CDC | T |  | 9.4 | 7.5-11.6 | 3.4 | 2.2-4.8 |  |  |
|  |  |  |  |  | WHO | T |  | 8.8 | 7.0-11.0 | 7.5 | 5.8-9.5 |  |  |
|  |  |  |  |  | National | T |  | 2.4 | 1.5-3.7 | 1.5 | 0.8-2.6 |  |  |
| Mirzaeian S et al(218) | Local | 2013 | Najafabad- U | 15-18 | IOTF | F | 550 |  |  |  |  | 25.6 | 22.0-29.5 |
| Khashayar P. et al(219) | NationalCASPIAN III | 2013 | 23 provinces | 10-18 | CDC | T | 5738 |  |  |  |  | 17.7 | 16.7-18.7 |
|  |  |  |  |  |  | M | 2863 |  |  |  |  | 19.9 | 18.5-21.4 |
|  |  |  |  |  |  | F | 2875 |  |  |  |  | 15.5 | 14.2-16.9 |
| Kelishadi R et al(220) | NationalCASPIAN IV | 2013 | 30 provinces-U | 6-18 | WHO | T | 13486 | 9.7 | 9.2-10.2 | 11.9 | 11.3-12.4 |  |  |
|  |  |  |  |  |  | M | 6846 | 9.3 | 8.6-10.0 | 13.6 | 12.8-14.4 |  |  |
|  |  |  |  |  |  | F | 6640 | 10.1 | 9.4-10.8 | 10.1 | 9.4-10.8 |  |  |

**Legend: n:** number, **CI:** confidence interval, **T:** total, **M:** male, **F:** female, **U:** Urban, **R:** Rural, CI 95%

IOTF, the International Obesity Task Force; CDC, the Center for Disease Control and Prevention; NCHS/WHO,The National Center for Health Statistics/World Health Organization; NHS, National Health Survey; TGLS, Tehran Lipid and Glucose Survey; CASPIAN, Childhood and Adolescence Surveillanceand Prevention of Adult Non-communicable Disease; GSHS-WHO, Global school based health survey questionnaire of the World Health Organization.
