# Supplementary material for: Prevalence of obesity and overweight in adults and children in Iran; a systematic review
Source: J Diabetes Metab Disord. 2014 Dec 23;13:121. doi: 10.1186/s40200-014-0121-2 (PMC4301060; doi:10.1186/s40200-014-0121-2)
Supplement: Additional file 3: Table S3. — A summary on the prevalence of overweight and obesity in Iran. [file 40200_2014_121_MOESM3_ESM.docx]

|  | Level of study | Gender | Overweight prevalence reports | | | | Obesity prevalence reports | | | |
| --- | --- | --- | --- | --- | --- | --- | --- | --- | --- | --- |
|  |  |  | Lowest prevalence (95% CI) | Location& reference | Highest prevalence (95% CI) | Location& reference | Lowest prevalence (95% CI) | Location& reference | Highest prevalence (95% CI) | Location & reference |
| Adult | National | T | 27.0 (26.8-27.1) | N ^(40)^ | 38.5 (37.2-39.8) | N^(35)^ | 12.6 (12.2-13.0) | N^(109)^ | 25.9 (24.9-26.8) | N^(52)^ |
|  |  | M | 14.4 (14.2-14.5) | N^(40)^ | 39.4 (36.3-42.5) | N ^(110)^ | 10.2 (8.4-12.2) | N^(110)^ | 26.0 (25.8-26.1) | N^(40)^ |
|  |  | F | 32.4 (32.0-32.9) | N^(2)^ | 38.2 (36.5-39.9) | N ^(35)^ | 11.0 (10.8-11.1) | N ^(40)^ | 34.0 (32.0-36.0) | N^(52)^ |
|  | Sub-national | T | 12.8 (9.2-17.3) | Shiraz ^(101)^ | 76.4 (75.1-77.6) | Tehran ^(48)^ | 2.4 (1.0-4.9) | Shiraz^(101)^ | 35.4 (31.4-39.6) | Rasht^(58)^ |
|  |  | M | 10.9 (8.8-13.2) | Shiraz ^(107)^ | 70.1 (67.9-72.1) | Tehran^(48)^ | 2.6 (0.7-6.5) | Shiraz^(101)^ | 38.0 (36.4-39.6) | Golestan^(64)^ |
|  |  | F | 9.6 (5.2-15.9) | Shiraz^(101)^ | 80.9 (79.3-82.4) | Tehran ^(48)^ | 2.2 (0.4-6.3) | Shiraz ^(101)^ | 52.8 (46.7-59.0) | Rasht^(58)^ |
| < 18 | National | T | 5.0 (4.5-5.5) | N ^(207)^ | 13.5 (13.4-13.6) | N^(34)^ | 3.2 (3.0-3.4) | N^(188)^ | 11.9 (11.3-12.4) | N ^(220)^ |
|  |  | M | 6.2 (5.5-7.0) | N ^(207)^ | 10.5 (10.0-11.0) | N^(188)^ | 3.3 (2.8-3.9) | N^(207)^ | 13.6 (12.8-14.4) | N ^(220)^ |
|  |  | F | 8.1 (7.7-8.5) | N^(188)^ | 10.1 (9.4-10.8) | N^(220)^ | 2.7 (2.4-3.0) | N^(188)^ | 10.1 (9.4-10.8) | N^(220)^ |
|  | Sub-national | T | 2.4 (1.5-3.7) | Zabol^(217)^ | 67.1 (63.3-70.8) | Isfahan ^(135)^ | 0.6 (0.3-1.1) | Kerman ^(169)^ | 27.7 (25.4-30.0) | Gorgan^(154)^ |
|  |  | M | 3.7 (3.1-4.4) | Birjand^(153)^ | 70.0 (64.6-75.0) | Isfahan ^(135)^ | 1.0 (0.4-2.0) | Kerman ^(169)^ | 28.9 (25.6-32.4) | Gorgan^(154)^ |
|  |  | F | 1.5 (0.5-3.2) | Ahwaz ^(156)^ | 64.2 (58.7-69.5) | Isfahan ^(135)^ | 0.1 (0.0-0.6) | Kerman ^(169)^ | 26.5 (23.3-29.8) | Gorgan^(154)^ |

**Table S3.** A summary on the prevalence of overweight and obesity in Iran.

**Legend: n:** number, **CI:** confidence interval, **T:** total, **M:** male, **F:** female, **N:** national.
